# Human infection or lab artifact: will the real XMRV please stand up?

**DOI:** 10.1186/1742-4690-8-S1-A241

**Published:** 2011-06-06

**Authors:** Robert H Silverman

**Affiliations:** 1 Department of Cancer Biology, Lerner Research Institute, Cleveland Clinic, Cleveland, Ohio, USA

## 

Xenotropic murine leukemia virus-related virus (XMRV) was first identified in 2005 in a study of human prostate cancer patients with genetic variants of the antiviral enzyme, RNase L. Subsequent investigations in North America, Europe and Asia have either observed or failed to detect XMRV in patients [prostate cancer, chronic fatigue syndrome-myalgic encephalomyelitis (CFS-ME), immunosuppressed with respiratory tract infections] or normal, healthy control individuals. Among the confounding factors are the potential for lab contamination with similar or identical viruses or viral sequences originating in mice. In some studies, relatively contamination-resistant methods (e.g. IHC, FISH, and antibody detection) suggest that either XMRV or a similar type of virus is present in some patients. Evidence for and against genuine infections of humans with this intriguing virus (and/or related viruses) will be discussed.

